# The Effects of COVID-19 and Quarantine Measures on the Lifestyles and Mental Health of People Over 60 at Increased Risk of Dementia

**DOI:** 10.3389/fpsyt.2020.578628

**Published:** 2020-10-14

**Authors:** Simona Gabriella Di Santo, Flaminia Franchini, Beatrice Filiputti, Angela Martone, Serena Sannino

**Affiliations:** ^1^Laboratory-Service of Epidemiology and Clinical Research (LASERC), IRCCS Fondazione Santa Lucia, Scientific Institute for Research, Hospitalization and Healthcare, Rome, Italy; ^2^Department of Systems Medicine, Università degli Studi di Roma 'Tor Vergata', Rome, Italy

**Keywords:** COVID-19, quarantine, mild cognitive impairment, subjective cognitive decline, lifestyle changes, depression, apathy, anxiety

## Abstract

**Background:** The lockdown strategies adopted to limit the spread of COVID-19 infection may lead to adopt unhealthy lifestyles which may impact on the mental well-being and future risk of dementia. Older adults with mild cognitive impairment (MCI) or subjective cognitive decline (SCD) may suffer important mental health consequences from measures of quarantine and confinement.

**Aims:** The study aimed to explore the effects of COVID-19 and quarantine measures on lifestyles and mental health of elderly at increased risk of dementia.

**Methods:** One hundred and twenty six community-dwelling seniors with MCI or SCD were phone-interviewed and assessed with questions regarding variables related to COVID-19 pandemic, lifestyle changes and scales validated for the assessment of depression, anxiety, and apathy.

**Results:** The sample included 55.6% patients with MCI and 56 people with SCD. Over 1/3 of the sample reduced their physical activity and nearly 70% reported an increase in idle time. Adherence to the Mediterranean diet decreased in almost 1/3 of respondents and over 35% reported weight gain. Social activities were abolished and 1/6 of participants also decreased productive and mental-stimulating activities. 19.8% were depressed, 9.5% anxious, and 9.5% apathetic. A significant association existed between depression and living alone or having a poor relation with cohabitants and between anxiety and SCD, cold or flu symptoms, and reduction in productive leisure activities.

**Conclusions:** Seniors with SCD and MCI underwent lifestyle changes that are potentially harmful to their future cognitive decline, even if, with the exception of leisure activities, they do not appear to be cross-sectionally associated with psychiatric symptoms.

## Background

The pandemic emergency linked to the spread of the new coronavirus disease COVID-19, led the Italian Government to adopt extreme measures of social distancing, which paralyzed the economy, the society and the daily life of thousands of people (1). The restrictive rules involved the whole population, with particular emphasis on older people and people with pre-existing medical conditions, since these individuals are extremely at risk of developing a Severe Acute Respiratory Syndrome (SARS), hospitalization, and death. Therefore, the indications contained in the DPCM of the 8th March 2020, made an “express recommendation to all people who are elderly or suffering from chronic or multi-morbid diseases […] to avoid leaving their home out of cases of strict need (2).”

COVID-19 can have direct and indirect effects on physical and mental health of the aged people. The SARS-CoV-2 virus which causes COVID-19 may affect central and peripheral nervous system (3, 4), having potential effects on the development and progression of neurodegenerative diseases (5). SARS-CoV-2 may also affect the cells of the intestinal mucosa, triggering intestinal inflammation and dysbiosis and potentially causing short and long-term alterations of gut microbiota, which have demonstrated strong associations with, neuroinflammation and neurodegenerative diseases (6, 7).

In order to reduce the spread of infection and optimize the management of the COVID-19 pandemic, some aspects of health management were modified: medical visits, non-urgent surgery and rehabilitative interventions were suspended, reduced, or post-poned; part of the visits were conducted by telephone or with the aid of telematic instruments. These changes may affect the management of elderly patients, who might encounter difficulties related to the modification of their routines and/or to the use of tools with which they are not familiar, and of patients with multiple comorbidities, who need integrated and continuous care, periodic symptoms monitoring, and readjustment of drug treatments. Moreover, elderly people and patients with multimorbidity may not access the medical visits for fear of being infected by COVID-19.

The lockdown measures in Italy led also to the closure of the day centers, offices of voluntary associations, churches, parishes, gyms, elderly universities, and other meeting places for seniors. Social disconnection is a risk factor for incident dementia, determining an increased risk of depression and anxiety for elderly people (8). Retrospective studies on the SARS epidemic in 2003 observed an increase in suicide rates between seniors during the epidemic period (9) and an online survey conducted last February in the regions of south-western China by Lei et al. (10), found that the inhabitants of the areas subjected to quarantine for COVID-19 showed almost double prevalence of depression and anxiety compared to the residents of the regions where isolation measures were not applied. Depression, anxiety and other neuropsychiatric symptoms represent risk factors for the conversion to dementia (11, 12); these symptoms worsen the quality of life of patients, accelerate the progression of the disease and lead to institutionalization and to an increase of health costs (13). Furthermore, lockdown could affect disproportionately the mental health of old people, whom relatives contracted COVID-19, people who live alone and whose only social contacts take place outside home, and people who do not have close relatives or friends and rely on the support of voluntary services or social assistance (14).

It is also important to note that changes in lifestyles, physical activity, and nutritional habits have a significant impact on cardiovascular risk factors (15), that are important predictors of dementia. In fact, it has been estimated that about a third of Alzheimer's disease cases (AD)—the most common form of neurodegenerative disease—is attributable to modifiable risk factors, as low education, smoking, physical inactivity, presence of hypertension, obesity, diabetes, depression (16, 17). Modifiable risk factors play also an important role in the conversion from MCI to dementia (11, 18), therefore, any COVID-19 related changes in lifestyle might affect the progression of cognitive impairment.

Eating habits may change during quarantine due to reduced availability of products, restrictions on access to stores (as, for example, the need to queue outside every store to do groceries), the fear of the possible lack of food which leads to the purchase and the consumption of packaged and preserved food, the reduced intake of fresh foods and the transition to unhealthy foods, such as snacks, and hunger-breakers—which may lead to a weight gain and to a reduced intake of antioxidants (15).

Also a decrease in the amount of time spent being physically active might have negative consequence on cognition and mental health. In a recent literature review, Narici et al. (19) described the impact of sedentariness potentially associated to COVID-19 on human body at the level of muscular, cardiovascular, metabolic, endocrine, and nervous systems and on the basis of several models of inactivity, including bed or couch rest, and reduced number of steps. A few days of sedentary life are enough to induce muscular loss, damage to neuromuscular junction and fibers' denervation (neuromuscular integrity is strictly binded to mitochondrial function), insulin resistance, reduction in aerobic capacity, fat deposition and low-grade systemic inflammation. Indeed, mechanisms involving oxidative processes, neuroinflammation and apoptosis have long been studied under different neurological conditions, as stroke, Alzheimer's disease, Parkinson's disease, multiple sclerosis (MS) and Huntington's disease (HD). Inflammatory processes are known to be closely linked to depression, hypertension, hyperlipidemia, insulin resistance, diabetes mellitus (DM), and obesity, small vessels' diseases and atherosclerosis, which are the main risk factors for the main cerebrovascular and neurodegenerative diseases, including AD (20).

A recently published study reported associations between changes in healthy behaviors and psychological distress in Australian adults during the COVID-19 pandemic: particularly, the more important were the negative changes in physical activity, sleep, smoking and alcohol consumption, the higher was the increasing in depression, anxiety and distress scores (21). No study, until today, inquired associations between changes in lifestyle and mental health issues in older adults at increased risk of dementia.

Many institutional sources, as scientific societies, the World Health Organization (WHO), the Italian Health Ministry, and the National Institute of Health, promoted and shared guidelines and tutorials dedicated to citizens or healthcare workers, in order to promote healthy lifestyles and maintain mental health during the lockdown phase (22–25). Free psychological support services were also provided including the National toll-free number 800.833.833, to meet psychological needs of people under quarantine and reduce the mental distress associated with COVID-19 (26). However, aged people, and in particular those who suffer from cognitive decline, may not have the necessary mental abilities to access these services.

Until today, there are only few studies who evaluated the effects of COVID-19 pandemic and of the quarantine measure on the psychological well-being, and lifestyles of older people and, in particular, of those at risk of cognitive decline.

Two studies observed that older people showed less COVID-19 outbreak-related emotional distress than younger ones, a more optimistic outlook and better mental health (27, 28). On the other hand, these researches enrolled seniors without cognitive impairment. It was observed that the COVID-19 related confinement aggravated the behavioral and psychological symptoms of community-dwelling older adults with mild cognitive impairment (MCI) or mild dementia, with agitation, apathy, and aberrant motor activity being the most affected symptoms (29, 30). A phone-based survey conducted by Goodman-Casanova et al. (31) in order to explore the well-being and the physical and mental health impact in community-dwelling older adults with MCI or mild dementia during the quarantine also showed that 46.1% participants reported negative experiences, such as fear of become infected or infecting family members, frustration and boredom involving not being able to take part in daily activities, loss of usual routine and social isolation. However, this latter did not assess mental health using validated scales and did not investigate whether there were any associations between lifestyle changes and negative experiences.

The aim of this observational study was to evaluate the effects of the COVID-19 outbreak and related infection control measures on the mental health and the lifestyles of older people at risk of dementia. In detail, we aimed to explore and analyse: (1) the reported changes in physical activity, leisure activities, smoking habits, caffeine and alcohol intake, eating behaviors, and in particular adherence to the MD during lockdown; (2) the presence of mental health issues, and in particular of depression, anxiety and apathy, according to validates scales. Another aim of this study was to identify (3) factors occurring during the COVID-19 pandemic which could be associated to the presence of depressive, apathetic, anxious symptoms.

## Methods

### Study Design and Description

This cross-sectional observational study included community-dwelling seniors ≥ 60 years of age with Mild Cognitive Impairment or Subjective Cognitive Decline who were enrolled in a randomized controlled trial (GR-2013-02356043, co-financed by the Italian Ministry of Health) aimed to assess the effectiveness of a 12-week intervention of cognitive stimulation and/or physical exercise in preventing dementia or cognitive and functional decline. Since GR-2013-02356043 was temporarily suspended due to the restrictions caused by COVID-19 pandemic, from April 21st to May 7th participants were contacted and interviewed by phone by trained psychologists. Due to the exceptional situation, informed consent to the interview and the use of the data collected during the GR-2013-02356043 study was provided orally or by SMS or e-mail. The study and its amendment were approved by the Ethic Committee of the IRCCS Fondazione Santa Lucia of Rome.

### Sample Characteristics

Inclusion criteria were: age ≥ 60 years; having undergone the last study visit in the preceeding 18 months; absence of a significant functional impairment in the last study visit, that was operationalized as a score <9 in the Functional Assessment Questionnaire (FAQ) or as a loss <20% in the Instrumental Abilities of Daily Living (IADL); diagnosis of MCI according to the International Working Group criteria (32) and cognitive impairment operationalized as a MMSE score ≥ 20 and ≤ 26 (or ≤ 28 for participants with 16 or more years of education) or as a score under the normative cut-off in at least one domain-specific cognitive test from an extensive neuropsychological battery (33). Participants with a diagnosis of Subjective Cognitive decline according to the International Working Group on SCD criteria (34), perception of a worsened cognitive efficiency, MMSE > 26 (or > 28 if 16 or more years of education) and absence of impairment in domain-specific neuropsychological scores were also included.

Exclusion criteria were diagnosis of dementia, presence of significant functional impairment, history of cerebrovascular or neurologic disease, drug or alcohol abuse, major psychiatric disease, presence of manifest sensory and motor deficits, contraindications to physical exercise, being unable—according to the caregiver's opinion—to perform the phone interview and inability to provide informed consent.

### The Survey

A structured questionnaire was specifically built with Google Forms (Google LLC) by the Epidemiology and Clinical Research Laboratory (LASERC) of IRCCS Fondazione Santa Lucia, in order to facilitate the insertion of data during the telephone interview and minimize the possibility of imputing incorrect data. The survey included 8 sections:

(1) Sociodemographic, anamnestic, lifestyle, and clinical data: information was collected about height, weight, weight changes during quarantine, living conditions (alone, with others), quality of relation with co-habitants (for participants not living alone), house size and presence of external spaces such as balconies and gardens, pet-ownership, possible comorbidities as hypertension, diabetes, cardiovascular diseases and other morbidities, psychiatric diagnoses, pharmacological treatment, quality of sleep, sleep changes since the beginning of the lockdown.
(2) Cognitive status was assessed through a phone version of the Mini Mental State Examination (Itel-MMSE) (35). Itel-MMSE is a validated Italian tool which shows strong correlations with paper-and-pencil MMSE (*r* = 0.85 in the whole sample and *r* = 0.77 in MCI subjects) (36) and it is predictive of domain-specific cognitive test performances (37). A regression equation allows to convert Itel-MMSE scores into MMSE (36) score. Participants who scored ≤ 17 were excluded from further analyses, since it was not possible to ascertain that they were not too cognitively compromised to appropriately understand and answer the questions.
(3) The Functional Assessment Questionnaire (FAQ) (38), the Basic Activities of Daily Living (BADL) and the Instrumental Activities of Daily Living (IADL) (39) were administered to evaluate functional independence. A FAQ cut-point of 9 was adopted to define the presence of clinically relevant functional impairment (40, 41) while IADL and BADL have no cut-off scores.
(4) COVID-19 and health: participants were asked to specify if they received a diagnosis of COVID-19, pneumonia, influenza, or if they had cold or flu symptoms since the 1st of February; if they contacted emergency numbers and/or their GP in presence of respiratory or any other kind of symptoms; if isolation was recommended to them; if they underwent oropharyngeal swab; if they were hospitalized for COVID-19 or other respiratory disease; if they knew people who got infected with SARS-CoV-2; if they had had any contact with people who got infected with SARS-CoV-2; if infected people were hospitalized/received intensive care/died due to COVID-19; presence of any changes, due to COVID-19, in the services they received by the health system, municipality, voluntary associations and other institutions.
(5) The emotional impact of the COVID-19 pandemic: participants were asked the reason why they started auto-isolation (ministerial decree, coming back from a “red zone,” personal decision before the decree, other); to quantify the impact of quarantine on their daily routine (none/slight/moderate/extreme); to specify changes of greater impact; the frequency and reasons for leaving home during the quarantine; engagement and frequency of violations of restrictive norms; the possibility of talking about their feelings and delegating some needs to relatives/friends/neighbors, to quantify any concerns about COVID-19 epidemic (none/slight/moderate/extreme), time they spent in informing and talking about the pandemic, concerns related to the influence of the pandemic on their health and their's family members'; presence/absent of persistent sadness, irritability and disengagement. Validated questionnaires were also used: the Geriatric Depression Scale-5-item (GDS-5) (42); the Apathy Evaluation Scale (AES) and the General Anxiety Disorder-7 (GAD-7) (43) were administered to screen depression, apathy and anxiety symptoms, respectively:
  - The GDS-5 is a short questionnaire investigating satisfaction with life, social withdrawal, feelings of emptiness/boredom, helplessness, and worthlessness. A point is assigned to the presence of each of these 5 items, resulting in a global GDS-5 score ranging from 0 to 5, with a cut-off score of 2.
  - The 18 questions of AES inquire 3 domains of apathy (decrease in goal-directed behaviors, reduction of goal-related thoughts, emotional indifference). Each item is scored on a 4-point Likert Scale, with overall AES scores ranging from 18 to 72, with a cut-off score of 38. Higher scores reflect more severe apathy.
  - The GAD-7 is a 7-item self-rated scale which describes the most salient diagnostic features of Generalized Anxiety Disorder (GAD). Each item is rated on a 4-point Likert Scale (0 = not at all to 3 = nearly every day). A cut-off score of 10 provides the best sensitivity and specificity for GAD diagnosis.

Other questions included the impact of information about COVID-19 on their feelings; having felt the need to consult freely available psychological services; having contacted the freely available psychological services; and whether they actually did.

(6) Physical activity was assessed with a modified version of the “International Physical Activity Questionnaire—short form” (IPAQ-SF) (44). IPAQ-SF records the individual's activity according to four intensity levels: (1) vigorous-intensity activity, such as aerobics, (2) moderate-intensity activity, such as leisure cycling, (3) walking, and (4) sitting or laying. The time spent in each activity level can be converted into Metabolic Equivalent of Task (MET) values, to obtain an index of the amount of the individual's total energy expenditure. A cut-point of 600 MET/week, roughly corresponding to 150 min of moderate intensity activity, was adopted to classify participants as physically active/inactive. Participants were asked to evaluate their physical activity levels via IPAQ during the last week before the phone call and during the last week before the lockdown.
(7) Food habits were assessed using the Mediterranean Diet Adherence Screener (MEDAS) (45), a 14-item questionnaire requesting participants to report food habits (consumption of olive oil and greater consumption of white meat, compared to red meat) and frequency of 11 consumption/amount of 12 main foods related to the Mediterranean Diet. Each of the 14 MEDAS items is assigned a score of 0 or 1, according to predetermined criteria. The maximum overall score is 14. Participants who obtained a MEDAS score <9 were classified as non-adherent to the Mediterranean diet. Participants were also asked to provide information about any changes dietary changes consequent to the lockdown, on tobacco, alcohol, and caffeine consumption and changes in consumption since the beginning of the lockdown were also inquired.
(8) A self-report questionnaire was created to investigate participation in 16 cognitively stimulating leisure activities or hobbies. Social and leisure activities were grouped following the classification adopted in the Kungsholmen Project (46). Mental stimulating activities consisted of reading books/newspapers, doing puzzle games like crosswords, card solitaires, sudoku, or others; singing; keeping informed about—or attending—economic, social, politic, or other news or events. Social activities included traveling; going to the cinema, theater concerts, or art exhibitions; playing cards/games with other people; volunteering/charitable activities; meeting relatives and friends. Productive activities included housekeeping, cooking, bricolage, collecting; writing; knitting or embroidery; painting, drawing or photographing; gardening; others. Recreational activities included watching television, movies, concerts, or theatral plays on the internet and listening to music.

### Statistical Analyses

Data were collected, preserved and analyzed in compliance with the applicable privacy rules. All the data were tabulated in a Google Sheet file.

The statistical analyses were performed with IBM SPSS ver. 20 (IBM, Chicago, IL, USA). GDS-5, GAD-7, AES, IPAQ, and MEDAS scores where dichotomized according to the previously described cut-points. A first description of the epidemiological characteristics of the sample was provided—data are represented as absolute frequencies and percentages (%) for categorical variables, as average ± standard deviation for continuous normally distributed variables or as median and interquartile range [IQR] for continuous not normally distributed variables; the Shapiro–Wilk test was performed in order to evaluate the normality of distributions.

The Pearson or Spearman correlation coefficients were then calculated in order to evaluate the correlation between continuous variables. Chi square test was used to assess the association between categorical variables. The McNemar test was calculated to inquire the difference between categorical variables before and after the COVID-19 lockdown. *T-*Test and McNemar *U-*test were performed to verify the presence of any difference between groups in continuous scores. Significance was set for a *p* < 0.05.

Univariate logistic regression analyses were performed to evaluate the association of variables—i.e., age, instruction, sex, group (MCI vs. SCD), Itel-MMSE score, pluripathologies, overweight, weight changes, smoking, changes in smoking habits, alcohol consumption, changes in alcohol consumption, caffeine, increased caffeine intake, living alone, quality of relationship with cohabitants, symptoms of cold or flu, knowing people with covid-19, leaving home at least once a week, personal decision to start quarantine, perceived impact of quarantine (high-moderate vs. fair-absent), presence of balcony/garden in their home, extent of concerns regarding COVID-19 pandemic, time taken to inquire about COVID-19, time taken to talk about COVID-19, extent of concerns for their health or that of their family members, IPAQ at least 600 MET/week, IPAQ decreased physical activity, adherence to the Mediterranean diet according to MEDAS score, dietary changes, variation in leisure activities—with the presence of depression in GDS-5, apathy in AES and anxiety in GAD-7. The association between being/not being anxious or apathetic or depressed was also assessed.

Finally, variables found to be statistically significant (at *p* < 0.1) in the univariate analyses were included in conditional multiple logistic models in order to determine the continuous or categorical variables which independently associated with the presence of depression, anxiety or apathy.

### Ethical Aspects

The study protocol was prepared in full application of Good Clinical Practice (GCP) guidelines for observational studies and of the Declaration of Helsinki for clinical trials in humans. The study was approved by the Ethic Committee of the IRCCS Fondazione Santa Lucia. Researchers made phone contact in respect of individual autonomy, and in compliance with current privacy regulations.

## Results

### Sociodemographic and Clinical Characteristics of the Sample

One-hundred seventy-six seniors at risk were contactable, who had been evaluated at LASERC in the previous 18 months. Five of them (2.84%) were unavailable and 40 (22.73%) refused to be interviewed; according to the caregiver's opinion, 3 (1.70%) were unable, due to cognitive impairment, to complete the interview. Therefore, 128 seniors were interviewed. Two of them (1.56%) were excluded from subsequent analyses because they had obtained an Itel-MMSE score <17. The final sample therefore consisted of 126 participants (71.59% of the interviewable seniors at risk) aged between 60 and 87 years (mean age = 74.29 ± 6.51 years); the sample was mainly composed of females (81.00%) and included 70 (55.55%) patients with MCI and 56 people with SCD (Table 1A). The interviewees obtained a median Itel-MMSE score of 21 (IQR = 2) and a median FAQ score of 0 (IQR = 1) No differences were observed between the cognitive and functional scores of participants who, in the last visit, were classified as MCI or SCD (Table 1B). Although the entire sample, with the exception of one interviewee, was generally independent in carrying out the instrumental activities of daily life, 5.55% of the respondents reported they needed to be helped in some higher cognitive-demanding tasks or to delegate.

**Table 1:** Sociodemographic, clinical, cognitive, and functional characteristics of the sample, divided by diagnosis.

	**SCD**	**MCI**	**Total**
**A. Demographics**			
Cases	56 (44.4)	70 (55.6)	126 (100)
Females	47 (83.9)	55 (78.6)	102 (81.0)
Age (years)	74.39 ± 6.38	74.20 ± 6.66	74.29 ± 6.51
Education (years)			
**B. Cognitive and functional status**			
*MMSE score (last visit)*	*27.55 [3.07]*	*26.78 [2.10]*	*27.30 [2.40]*
Itel-MMSE score	21.50 [1.50]	21.00 [2.00]	21.00 [2.00]
FAQ score	0.00 [0.50]	0.00 [2.00]	0.00 [1.00]
**C. Clinical data**			
Overweight/obesity	25 (44.6)	37 (52.9)	62 (49.2)
Hypertension	27 (48.2)	41 (58.6)	68 (54.0)
Hyperlipidemia	28 (50.0)	34 (48.6)	62 (49.2)
Diabetes	5 (8.9)	8 (11.4)	13 (10.3)
Cardiovascular dis.	13 (23.2)	27 (38.6)	40 (31.7)
Musculoskeletal dis.	10 (17.9)	11 (15.7)	21 (16.7)
Thyroid dis.	14 (25.0)	22 (31.4)	36 (28.6)
Autoimmune dis.	2 (3.6)	7 (10.0)	9 (7.1)
Pre-existing respiratory dis.	3 (5.4)	8 (11.4)	11 (8.7)
Other dis.	11 (19.6)	14 (20.0)	25 (19.8)
2 or more comorbidities	38 (67.9)	54 (77.1)	92 (73.0)
Regular drug consumption	52 (92.9)	63 (90.0)	115 (91.3)
Poor sleep quality	47 (83.9)	56 (80.0)	103 (81.7)
Worsened sleep	3 (5.4)	4 (5.7)	7 (5.6)
**D. Living conditions**			
Lived alone	15 (26.8)	21 (30.0)	36 (28.6)
Absence of external openings at home	9 (16.1)	10 (14.3)	19 (15.1)
Poor relation with cohabitants	9 (22.0)	6 (12.2)	15 (16.7)
Had pets	8 (14.3)	20 (28.6)	28 (22.2)

*Results are reported as absolute frequencies and percentages (in brackets) for categorical variables, as average ± standard deviation for continuous normally distributed variables or as median and interquartile range [IQR] for continuous not normally distributed variables. In italics, the statistically significant differences between participants with SCD and MCI, with p-level < 0.05, are reported*.

#### Clinical Characteristics

One hundred and one participants (96.03%) have at least one comorbidity among hypertension (53.97%), hyperlipidemia (49.21%), diabetes (10.32%), cardiovascular diseases (31.75%), musculoskeletal disorders (16.67%), thyroid dysfunction (28.57%), autoimmune diseases (7.14%), pre-existing respiratory illnesses (8.73%) or others (31.75%); 72.23% aged people had multi-morbidity; 105 (91.27%) regularly assumed one or more medicines. Although a slightly higher number of seniors with MCI had clinical complaints, there were no statistically significant differences in the proportions of SCD or MCI participants with clinical conditions and with multicomorbidity (Table 1C).

One hundred and three participants (81.75%) reported good/fair sleep quality; 7 (5.55%) reported a deterioration in sleep quality after the start of the lockdown, with no statistically significant differences between MCI and SCD participants (Table 1C).

#### Living Conditions

Thirty-six participants (28.57%,) lived alone, while the remainder shared their home with one or more co-habitants (in 95.55% of cases, spouses and/or children); 4.8% declared that they had changed their living situation in order to deal with the quarantine, by welcoming relatives into their home or by moving to their relatives' houses (Table 2C); one participant with SCD reported that he had gone to live alone, to avoid the transmission of the infection to his relatives. The relationship with the cohabiting people was declared good or fair from the clear majority of the sample, with only 2 cases reporting a poor relationship. Nineteen aged ones (15.08%) stated that their houses did not have a garden, a terrace or any other type of external opening that would allow them to go outside without leaving home.

**Table 2:** Clinical information regarding the health of the participants and their acquaintances, and data concerning the quarantine and facilities available during it.

	**SCD**	**MCI**	**Total**
**A. COVID-19 and health status**			
Cold or flu symptoms	17 (30.4)	12 (17.1)	29 (23.0)
Referred to the physician/emergency services	5 (29.4)	1 (8.3)	6 (20.7)
Insulation recommended	1 (20.0)	0 (0.0)	1 (16.7)
Received COVID-19 diagnosis	0 (0.0)	0 (0.0)	0 (0.0)
*New drugs prescription*	*8 (14.5)*	*2 (2.9)*	*10 (8.0)*
**B. COVID-19 among known people**			
Knew COVID-19 cases	4 (7.1)	7 (10.0)	11 (8.7)
Had physical contact with them	1 (25.0)	2 (40.0)	3 (33.3)
Friends/relatives hospitalized for COVID-19	1 (25.0)	2 (40.0)	3 (33.3)
Friends/relatives dead for COVID-19	1 (25.0)	1 (20.00)	2 (22.2)
High/moderate distress associated with it	2 (50.0)	2 (40.0)	4 (44.4)
**C. Quarantine**			
Started spontaneously	15 (26.8)	20 (28.6)	35 (27.8)
Of high/moderate impact on daily routine	48 (85.7)	52 (74.3)	100 (79.4)
Violated for unauthorized reasons	4 (7.1)	4 (5.7)	8 (6.3)
Determined changes in living conditions	5 (9.1)	2 (2.9)	7 (5.6)
**D. Facilities**			
Home delivery from volunteers	2 (3.6)	0 (0.0)	2 (1.6)
Called COVID-19 related numbers	0 (0.0)	0 (0.0)	0 (0.0)
Had someone to turn to for help	50 (89.3)	67 (95.7)	117 (92.9)
Had someone to talk with about his/her feelings	55 (98.2)	65 (94.2)	120 (96.0)

*Results are reported as absolute frequencies and (percentages). In italics, the statistically significant differences between participants with SCD and MCI, with p-level < 0.05, are reported*.

Twenty-eight participants (22.22% of the sample, 6 of them living alone) had one or more pets (in 100% of cases dogs or cats). No differences were reported by participants with SCD or MCI regarding living conditions (Table 1D).

### COVID-19 and Health Status

Twenty-nine (23.01%) had cold or flu symptoms since the second half of february, and 6 (20.69% of the symptomatics) contacted the doctor and/or emergency numbers; 2 of them (33.33%) received diagnosis of flu. Isolation was recommended to 1 participant, who did not undergo oropharyngeal swab. Two asymptomatic seniors (1.59% of the sample) swabbed, with negative results, in order to undergo day-hospital interventions. Therefore, none of the participants were diagnosed with COVID-19. Ten seniors (7.94%) were prescribed new medicines during the COVID-19 emergency, in 8 (80%) cases medicines to treat cold or flu, in one case melatonin and in one lorazepam due to sleep disturbances and anxious symptoms that emerged after the lockdown. Significantly more seniors with SCD received new drug prescriptions than participants with MCI (Table 2A).

Eleven participants (8.73%) stated that they knew people who had contracted COVID-19 (Table 2B). Three 3 of the COVID-19 cases were hospitalized and 2 died during hospitalization. Four of the participants who knew or had contact with COVID-19 cases (36.36%) experienced symptoms such as soreness (2 cases, one of which also experienced difficulty breathing), running nose (1 participant), anxious symptoms such as tachycardia and tightness in the chest (1 respondent). Two of these 11 participants had physical contact with confirmed cases of COVID-19 and one with a suspected case of COVID-19 (Table 2B). None of the three cases was hospitalized or died and none of the 3 participants who had close contact with a confirmed/suspected case experienced flu-like or anxious symptoms.

The stress associated with knowing and/or having been in contact with a person affected by COVID-19 was assessed as moderate/high by 4/11 seniors (36.36%) and as low/absent by 3 (27.27%); 4 participants did not know how to answer, as they claimed to be unable to discriminate the amount of stress that was specifically associated with this condition from to the overall stress associated with the pandemic (Table 2B).

### The COVID-19 Related Quarantine

Ninety-two interviewees (73.02%) stated that they had started quarantine following the ministerial decree or after returning from a “red zone” (1 case); More than a quarter participants (26.98%) declare that they started the isolation spontaneously, before the official regulation (Table 2C).

Only 2 seniors (1.59%) declared that the Government provisions had had no effect on their daily routine. For everyone else, the lockdown had a big (33.33%), moderate (46.03%), or slight (18.25%) impact (Table 2C).

Slightly more than a responder in 10 (11.90%) reported that they never left home during the quarantine. The others went outside daily or almost daily (23.02%), several times a week (19.84%), once a week (20.63%), several times a month (17.46%) or less frequently (7.14%), for reasons permitted by the Ministerial Decree. Eight seniors (6.35%) admitted having violated the quarantine for unauthorized reasons such as meeting other people (37.50% of violations), leaving home beyond the allowed distance (50.00%) or others (25.00%) (Table 2C).

None of the interviewees stated that, before the COVID-19 emergency, they had received any type of home assistance from the Health System or the Municipality, or from voluntary associations. Two participants (1.60% of the 125 respondents) received facilities created to deal with the consequences of the pandemic (home delivery of medicines and groceries from volunteers). 117 (92.86%) seniors reported having other people available to whom they can ask for help in case of need, 7 (5.55%) claimed that they had no need to seek outside help and 2 (1.59%) admitted that they had no one to turn to, even if they needed (Table 2D).

One-hundred and twenty elders at risk (96.00% of 125 responders) reported that they had someone to turn to (family members, friends or other people) when they needed to talk about their feelings; 123 participants (98.40%) reported that they perceived no need having recourse to the free psychological public support services that were available to deal with the emotional impact of the pandemic; 2 seniors with SCD stated that they would have resorted to them, however they did not (Table 2D).

### Lifestyles, Behaviors, and Emotional Status During Quarantine

#### Lifestyles

Eighteen seniors (14.3%) were smokers (15.82 ± 7.86 cigarettes/day on the average). A third of them declared having smoked a higher number of cigarettes than before, since the beginning of the quarantine, while 2 reported having smoked less (Graph 1 reports percentages referred to the valid cases, i.e., participants who answered the question about smoke). Nobody started or stopped smoking after the lockdown. Non-significant differences were observed among MCI and SCD participants in the proportion of smokers (Table 3A) or of people who reported any variation in smoke (Chi-2, 1 df = 0.11 *p* = 0.744).

**Table 3:** Lifestyles, behaviors and emotional state during quarantine, divided by diagnosis.

	**SCD**	**MCI**	**Total**
**A. Lifestyles during quarantine**			
Smoke	8 (14.3)	10 (14.3)	18 (14.3)
Alcohol	23 (41.1)	33 (47.1)	56 (44.4)
Caffeine	45 (80.4)	61 (87.1)	106 (84.1)
Low physical activity (<600 MET/week)	23 (41.1)	37 (52.9)	60 (47.6)
Low adherence to MeDi diet	22 (40.0)	31 (44.3)	53 (42.4)
**B. Daily leisure activities during quarantine**			
Passive recreational	54 (98.2)	69 (98.6)	123 (98.4)
Mind-stimulating	40 (72.7)	56 (80.0)	96 (76.8)
Productive	48 (87.3)	61 (87.1)	109 (87.2)
Social	2 (3.6)	4 (5.7)	6 (4.8)
**C. Time spent for COVID-19**			
Time spent informing on media <30 min/day	15 (26.8)	22 (31.4)	37 (29.4)
<2 h/day	19 (33.9)	19 (27.1)	38 (30.2)
2+ h/day	22 (39.3)	29 (41.4)	51 (40.5)
Time spent talking about it <30 min/day	36 (64.3)	44 (62.9)	80 (63.5)
<2 h/day	12 (21.4)	15 (21.4)	27 (21.4)
2+ h/day	8 (14.3)	11 (15.7)	19 (15.1)
High/moderate influence of news on feelings	42 (75.0)	47 (68.1)	89 (71.2)
**D. Psycho-emotional status**			
Spontaneously declared high/moderate concern	46 (83.6)	58 (82.9)	104 (83.2)
Spontaneously declared being sad/depressed	17 (30.4)	17 (24.6)	34 (27.2)
Spontaneously declared being nervous/irritable	20 (35.7)	18 (25.7)	38 (30.2)
Spontaneous declared loss of interest	8 (14.3)	10 (14.3)	18 (14.3)
GDS-5 ≥ 2	13 (23.2)	12 (17.1)	25 (19.8)
*GAD-7 ≥ 10*	*9 (16.1)*	*3 (4.3)*	*12 (9.5)*
AES ≥ 38	3 (5.4)	9 (12.9)	12 (9.5)

*Results are reported as absolute frequencies and (percentages). In italics, the statistically significant differences between participants with SCD and MCI, with p-level < 0.05, are reported*.

Fifty-six interviewees (44.4%) reported regular alcohol consumption (Table 3A), on average 1.15 ± 0.69 alcoholic units (AU) per day during quarantine. During the lockdown, 7.0% of drinkers increased their alcohol consumption, 12.4% decreased it—among them, 2 (28.6%) declared having stopped drinking alcohol (Graph 1). Two participants declared having started drinking ½ glass of wine per day. Non-significant differences were observed among MCI and SCD participants in the proportion of drinkers (Table 3A) or of people who reported any variation in alcohol consumption (Chi-2, 1 df = 1.80 *p* = 0.180).

One-hundred and six participants (84.1%) reported drinking coffee or tea (Table 3A), on average 2.09 ± 1.02 cups/day. During the lockdown, caffeine consumption remained stable for most of them (84.9%), while 6.6% declared having increased and 8.5% stated having decreased the number of cups of coffee/tea per day (Graph 1 reports percentages referred to valid cases). Nobody started or stopped drinking caffeine during the lockdown. Non-significant differences were observed among MCI and SCD participants in the proportion of caffeine consumers (Table 3A) or of people who reported any variation in caffeine consumption (Chi-2, 1 df = 0.76 *p* = 0.384).

Forty-six participants (36.5% of valid cases and 43.4% of those that before the lockdown reached the recommended threshold of 600 MET/week) declared having decreased their physical activity to <600 MET/week, since the start of the lockdown; 69.60% of the sample reporting an increase in the time spent sitting or laying down (idle time). At the moment of the interview, half (52.4%) of the sample did not reach that threshold, with no significant differences between MCI and SCD participants (Table 3A), while, before the lockdown, only 25 interviewee (19.8%) had scored <600 MET/week. McNemar's test determined that there was a statistically significant difference in the pre- and post-quarantine proportions of participants above /below the recommended 600 MET/week threshold (*p* < 0.001). Non-significant differences were observed between MCI and SCD participants (Table 3A) in the proportions of people who reported any variation in physical activity (Chi-2, 2 df = 1.75 *p* = 0.416). However, 5 of the 25 respondents who, before the lockdown did not reach the recommended threshold of 600 MET/week, increased their physical activity levels to over 600 MET/week during quarantine, and 6 of them reported a decrease in idle time.

Forty-seven participants (37.6% of 125 respondents) reported that the quarantine had caused some changes in their nutritional habits. Of these, 19.2% reported to eat in higher amounts, 31.9% to eat more sweets, 12.8% to use more frequently preserved or frozen foods, 8.5% to have a less varied menu, 14.9% to eat in an unregulated or unhealthy way, 6.4% to eat more regularly/healthily, 2.5% to eat less, 17.0% other. However, 57.6% of the sample obtained MEDAS scores indicative of adequate adherence to the Mediterranean diet (Table 3A). Non-significant differences were observed among MCI and SCD participants in the proportion of people with adequate adherence to MD (Table 3A) or of people who reported any variation in dietary habits (Chi-2, 2 df = 0.75 *p* = 0.688).

During the quarantine, 35.7% of the sample reported having gained weight, and 11.1% declared having lost weight (Graph 1). At the moment of the interview, almost half of the participants (49.2%) were overweight or obese; and 2.4% were underweight. Non-significant differences were observed among MCI and SCD participants in the proportion of people with overweight/obesity (Table 3A) or of people who reported any weight change (Chi-2, 2 df = 0.82 *p* = 0.663).

One hundred and twenty-five participants completed the evaluation of the leisure activities (Table 3B). As expected, the whole sample declared having reduced their social activities since the start of the quarantine. However, 11.2% of the participants reported that they still engaged in social activities such as meeting with other people keeping the safety distance (mainly neighbors) or attending groups on online platforms at least once at week, without differences between SCD and MCI responders in the proportion of people engaging in social activities (Table 3A). The 58.1, 45.2, and 55.2% of the sample reported an increase in the time spent engaging in recreational, mind-stimulating and productive activities, respectively, while 5.6, 16.1, and 16.8%, respectively, declared carrying out these activities less frequently than before the lockdown. At the time of the interview, 76.8 and 96.0% of the interviewees, respectively, reported to practice mental activities at least daily and productive activities at least weekly. Non-significant differences were observed among MCI and SCD participants in the proportion of people which engaged in these activities (Table 3B) or of people who reported any variation in them (Recreational: Chi-2, 2 df = 2.29 *p* = 0.318; Mind-stimulating: Chi-2, 2 df = 2.81 *p* = 0.245; Productive: Chi-2, 2 df = 0.36 *p* = 0.834).

Variations in lifestyles after the lockdown in valid cases (i.e., in the participants who answered each question) are shown in Graph 1. Variables with ^*^ are reversed, so that red represents a potentially negative change in health and/or on the risk of dementia, and blue represents a positive one.

#### Behaviors

The estimated time spent in searching information about COVID-19 in the media was <30 min/day for 37 (29.4%) participants; <2 h/day for 38 (30.2%); and 2 or more h/day for 51 (40.5%). The estimated time spent in talking about COVID-19 with other people was <30 min/day for 80 (63.5%) participants; <2 h/day for 27 (21.4%); and 2 or more hours/day for 19 (15.1%). According to 19 seniors (15.2% of 125 respondents), the news about the coronavirus that they received from the media (TV, radio, newspapers, social networks, and others), had a great influence on their feelings. 56.0, 23.2, and 5.6%, respectively, reported that they were enough, little or not at all influenced by these informations (Table 3C).

#### Psycho-Emotional Consequences

The most important changes with the greatest emotional impact associated with the lockdown concerned the inability to meet children, grandchildren or other family members (41.3%) or friends (19.1%); attend meeting places, cinema, theater or dance hall (29.4%); leaving home, going out for a walk (24.6%); carry out the usual physical activity outside home or at the gym (23.8%); get help from a domestic worker (14.3%); cancellation of medical visits or physiotherapy treatments (5.6%); absence of human contact (5.6%); others (33.3%). Seven participants (5.6%) reported no substantial changes.

Forty people (31.8%) rated their level of concern associated with the COVID-19 pandemic as high, 64 (50.8%) as moderate, 16 (12.7%) as low and 5 (4.0%) as absent. Main concerns included the possibility of contracting COVID-19 (55.0%); the possibility that some family member fell ill with COVID-19 (53.3%); worries regarding the effect of the pandemic on their own health (50.8%) or on the health of their family members (35.8%) (e.g., difficulty in receiving adequate and timely treatment for their comorbidities due to the emergency); concerns about the personal/family economic or working situation (34.2%); concerns for the socio-economic future of the Nation (38.3%); other (19.1%).

The 11.1% of the sample declared themselves very concerned about their health or their family's health; 48.4, 23.8, and 16.7%, respectively, declare themselves quite, slightly and not at all worried.

More than a quarter participants (27.0%) declared themselves not worried at all that their health or their family's health may worsen during the pandemic; 45.2, 23.8, and 4.0%, respectively, declare themselves slightly, quite, and very concerned.

Thirty-four interviewees (27.2% of 125 respondents) declared that, since the start of the lockdown they had often felt sad, depressed, downcast, so much so that nothing could cheer them up. When evaluated with GDS-5, 25 (19.8%) participants obtained a score ≥ 2 (Table 3D). Depression was significantly associated with living alone or being in a poor relationship with cohabitants, low sleep quality and not owing a pet (Table 4).

**Table 4:** Univariate and multivariate analyses of factors which resulted associated with depression, anxiety and apathy.

	**Univariate analysis**				**Multivariate analysis**			
	**OR**	**95% CI inf**	**95% CI sup**	***p***	**OR**	**95% CI inf**	**95% CI sup**	***p***
**A. Depression**								
*Alone or poor relation*	4.19	1.64	10.68	0.003	*2.79*	*1.20*	*6.49*	*0.017*
Poor sleep quality	2.70	0.99	7.35	0.047	1.85	0.80	4.29	0.154
No pets	8.77	1.13	66.67	0.038	0.16	0.02	1.20	0.075
**B. Anxiety**								
*Subjective cognitive disorder*	4.28	1.10	16.64	0.036	*4.39*	*1.03*	*18.69*	*0.05*
*Cold/flu symptoms*	3.96	1.17	13.41	0.027	*4.01*	*1.13*	*14.24*	*0.03*
*Reduction in productive activities*	3.26	0.86	12.36	0.082	*4.42*	*1.10*	*17.76*	*0.04*
Time spent searching information	3.30	0.94	11.63	0.063	2.45	0.71	8.45	0.16
**C. Apathy**								
Alone or poor relation	5.14	1.32	20.06	0.018	3.73	0.96	14.45	0.057
Cold/flu symptoms	3.96	1.17	13.41	0.023	2.56	0.81	8.09	0.110
Non-adherence to MD	3.02	0.86	10.63	0.085	2.76	0.83	9.21	0.099
Reduction in productive activities	4.33	1.22	15.32	0.023	2.66	0.82	8.68	0.105

*In italics, the variables statistically associated with each psychological disorder in the multivariate analysis are reported, with p-level < 0.05. OR, odds ratio; 95% CI, 95% confidence interval; inf, inferior; sup, superior*.

Thirty-eight seniors (30.2% of the sample) reported feeling often irritated, nervous and getting angry easily. When evaluated with GAD-7, 47 (37.3%) participants scored ≥ 5 and 12 (9.5%) obtained scores indicative of at least moderate anxiety (Table 3D). Anxiety resulted associated with SCD, having had cold/flu symptoms, reduction in productive activities, and with high time spent searching information about COVID-19 on the media (Table 4).

Eighteen interviewees (14.3%) reported having lost interest in many of their activities, hobbies, or friends/relatives since the beginning of the quarantine; 12 (9.5%) participants were categorized as apathetic according to AES (Table 3D). Apathy associated significantly with living alone or being in a poor relationship with cohabitants, having had cold of flu symptoms, non-adherence to MD and reduction in productive activities.

### Multivariable Logistic Regression Analyses

In the multivariable logistic regression analyses (Table 1), depression resulted significantly associated with living alone or having a poor relation with cohabitants (OR: 2.79, 95% CI: 1.20–6.49); anxiety associated significantly with the presence of Subjective Cognitive Decline (OR: 4.39, 95% CI: 1.03–18.69, Table 1A), having had cold or flu symptoms (OR: 4.01, 95% CI: 1.13–14.24, Table 1B), and with a reduction in productive activities (OR: 4.41, 95% CI: 1.10–17.76). No significant associations were observed with apathy for variable that associated in the univariate analyses, when included in the multiple conditional model (Table 1C).

## Discussion

During COVID-19 outbreak, quarantine demonstrated an effective measure to prevent the further spread of the infection. However, it had negative effects that may hamper the psycho-physical well-being of people who are quarantined and it determined changes in lifestyles which might be associated with an increased future risk of dementia. To our knowledge, this is among the first studies evaluating the impact of the COVID-19 lockdown on lifestyle changes among seniors at increased risk of dementia and to analyse the association between variables related with the COVID-19 pandemic and depression, anxiety and apathy in this population.

**Figure 1:**
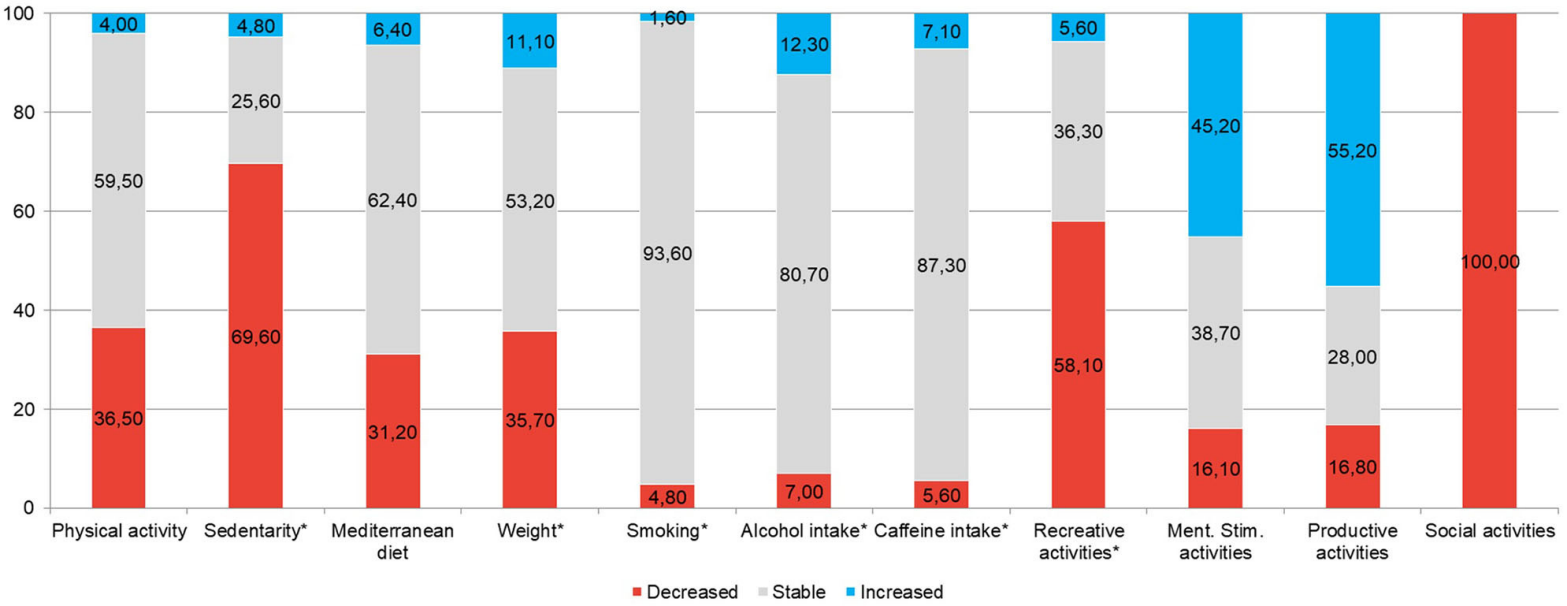
Lifestyle changes after the lockdown. The variable with * are reversed, so that red represents a potentially negative variation on health and/or on future risk of dementia, and blue represents a positive one.

Quarantine implied that over a third of the sample reduced their physical activity levels from over 600 MET/week to <600 MET/week. In addition, nearly 70% of the sample reported an increase in time spent sitting or lying down. Adherence to the Mediterranean diet also decreased in almost a third of respondents and over 35% reported weight gain. Minor changes were observed with respect to smoking or drinking alcohol or caffeine. As widely expected, the sample completely reduced social activities, but, at the same time, nearly 60% of seniors reported an increase in time spent in passive recreational activities, such as watching television or listening to the radio. Conversely, one in six elderly people at risk of dementia also decreased production and mental-stimulating activities (Figure 1) even if most of the sample, especially people with MCI, engaged in daily mental-stimulating activities. Changes toward increased sedentary lifestyle, overweight, unhealthy diet and lower engagement in non-passive recreational activities can increase the risk of dementia, since these variables have been consistently associated in middle age with a subsequent increased incidence of Alzheimer's disease and other dementias (16). An association, although weaker and non-invariable, also exists with an increased risk of dementia in people with MCI (11, 18) and recent evidence indicate that poor social interactions, small social networks, and low level of physical activity are correlated with depressive symptoms in community-dwelling seniors with MCI (47).

If it is true that the changes implied by the quarantine may be temporary and their effects lower compared to long-lasting lifestyles, it is equally conceivable that, at the end of the lockdown phase, many people will not return to their pre-pandemic “normal routine.” Unhealthy lifestyles adopted during lockdown could be maintained despite the reopening—for example, it is possible that people who before the lockdown used to go to the gym to exercise and who during quarantine stopped their training, will not start again, because of the fear of contagion or other reasons—similarly to what reported about MERS (Middle East Respiratory Syndrome), where it was observed that psychological difficulties associated to quarantine persisted for 4–6 months beyond the end of the restrictions in almost half of the people (48). Therefore, the use of quarantine as a measure of public health must consider the potential acute and chronic psychological effects of this procedure. In addition, the early assessment of the consequences of this measure on health conditions of the population at risk of dementia and the study of the strategies to limit this effect are particularly important (30).

Eighteen seniors of our sample (14.29%) were smokers. A third of them declared having smoked a higher number of cigarettes than before, since the beginning of the quarantine, while only 2 reported having smoked less. Moreover, most of our participants who, in the pre-quarantine were physically active, reduced time spent engaging in physical activity. Our results are in agreement with a recent web-based cross-sectional study (49) which showed an increased number of cigarettes per day among those who were smokers, and are in disagreement with those from a recent survey conducted by Di Renzo et al. (50) that showed reduced smoking habits and increased physical activity after the COVID-19 lockdown in a high proportion of Italian responders: authors hypothesized that their this results might be due to the fear, in Italians, of increased risk of respiratory distress and mortality from COVID-19 associated with smoke (51). Their sample was mainly composed of younger respondents without cognitive impairment. It has been hypothesized that people with cognitive decline may not be fully aware of the risks associated with the pandemic, and therefore less likely to adopt coping strategies. On the other hand, the proportion of smokers between participants with MCI and SCD in our sample is largely overlapping, so our results our results cannot be solely attributable to a lack of risk awareness. It is possible that the observed differences with respect to that survey are attributable to age differences in risk perceptions. Accordingly, the recent survey of Bruine de Bruin (28) observed that the fear of contracting the virus or of health consequences of the COVID-19 pandemic appears to decrease with increasing age.

Nevertheless, also a consistent part of our participants still implemented coping strategies such as increasing physical activity levels, improving their nutritional style, engaging in cognitively stimulating or productive activities, reducing smoking. Moreover, different from Di Renzo et al. (50), only 14.89% of our respondents reported greater tendency to eat unhealthy after the lockdown (less than half compared to 35.08% of their sample). However, this higher percentage may again reflect age differences: in fact, they reported higher appetite increase in younger people while our sample is made up of seniors ≥ 60 years of age. It has been shown that the adoption of healthy behaviors during quarantine may be useful to fight against the mental and physical consequences of COVID-19 quarantine, especially in older people. Our study did not reveal any association between lifestyle factors and GDS-5, GAD-7 scores o AES in our sample. Therefore, at least in the short term, maintaining an active lifestyle seems not to be protective against cognitive decline, depression or anxiety. However, we must consider the cross sectional nature of our study, which is a limitation that does not allow us to draw any definitive conclusions. It is therefore plausible that in the long term the seniors who engaged in active lifestyles will have a slower progression of their cognitive decline and a lesser probability that their mental health status will worsen.

Although Bruine de Bruin et al. (28) observed that older people experience less negative emotions than younger ones, we found that, since the start of the lockdown, thirty-four participants reported that they often felt so much sad, depressed or downcast that nothing could cheer them up. The 19.84% of our interviewees had a GDS-5 score indicative of depressive symptoms. Our results, showing a significant association between depression and living alone or having a poor relationship with the cohabitants, are in contrast with a recent cross-sectional study based on a national online survey in Spain conducted by García-Fernandez et al. (27) which did not show any relationship between loneliness and increase of depression in older adults. However, our results are consistent with another study conducted by phone-interviewing elders with MCI (31) and with the hypothesis that quarantine period affects mental health of older people who live alone and whose only social contacts take place outside home (14). Therefore, our results suggest that particular attention should be placed on social isolation for older people living alone or having bad relationships with family-members.

In our study, 30.16% reported feeling often irritated, nervous and getting angry easily. The scores obtained in GAD-7, showed that 37.30% of the participants scored ≥ 5 (mild anxiety) and 9.52% obtained scores indicative of clinically significant anxiety. These results are consistent with the study by Bruine de Bruin et al. (28). We found a significant association between anxiety and perceiving SCD: this could mean that people with SCD are more concerned about their cognitive status and their health, showing increased awareness. Increased anxiety is also associated with the presence of flu symptoms: considering their higher vulnerability, older people perceive the risk of contracting the virus and the manifestation of flu or COVID-19 symptoms, e.g., fever or cold, which inevitably increases the concern of a probable contagion. On the other hand, it is equally possible that anxious people are overly focused on their symptoms and emphasize signs of cognitive decline that are part of normal aging and physical symptoms of negligible severity. In fact, none of our participants had such severe flu or respiratory symptoms that they required hospitalization or performing the oropharyngeal swab. In addition, we found an association between anxiety and reduced productive leisure activities: we hypothesized that, probably, these people could not get away from their worries. However, it is also possible that the impossibility of dedicating to some productive activities, which were carried out outside the home before the lockdown, led to greater levels of anxiety. Again, the cross-sectional nature of the study does not allow us to verify any causal associations.

In a recent study, Beatriz Lara et al. (30) interviewed MCI and mild AD patients 5 weeks after the start of the lockdown by using the Neuropsychiatric Inventory (NPI) and the EuroQol-5D who were evaluated with the same scales a month before the lockdown. In both groups, symptoms related to apathy increased after a few weeks: comparing the presence of these symptoms before and after lockdown, they found that both in MCI and in AD patients apathy increased, although they did not observe changes in their quality of life. We found that 14.29% of the participants reported having lost interest in many of their activities, hobbies, or friends/relatives since the beginning of the quarantine; while 9.52% participants were categorized as apathetic according to AES. Univariate analyses revealed a significant association between apathy and no adherence to Mediterranean Diet, decrease of time in productive leisure activities, living alone or having a poor relation with relatives, however these associations lost significance in the multiple model.

Although a not negligible percentage of the sample reported the presence of psychiatric symptoms and/or of emotional consequences of the lockdown on their feelings, almost the whole sample did not feel any need to resort to the free psychological support services that were made available to counteract the emotional impact of the pandemic. The only two participants who thought to recurr to a call-center, desisted from doing so. Two seniors instead turned to their GP, who prescribed pharmacological treatments. This has possible implications regarding the strategies adopted at the public level to counteract the possible psycho-emotional consequences of the pandemic in elderly people at risk of dementia: although not in any way conclusive, the data available to us indicate that seniors with MCI or SCD tend to not think about and to not resort to call center services or unknown professionals in case of emotional distress, perhaps because they do not remember having this opportunity, perhaps because they prefer to turn to their known and trusted physicians or perhaps because they find themselves uncomfortable talking about their difficulties by phone instead of through a face-to-face conversation. In anticipation of a possible second wave of COVID-19 it would be advisable to strengthen the capacity of GP offices to take care of the psychological well-being of elderly patients.

### Limits

Beyond the cross-sectional nature of the study and the limited sample size, that warrants making any conclusive inference, participants were recruited among elderly that were included in a prevention programme, whose aim was reducing the risk to develop dementia, while we do not have any data about elderly who were not interested in taking part in the study. Moreover, almost a third of selected did not participate to the phone interview. Therefore, we should not overlook the presence of a potential selection bias. Furthermore, our sample includes only people with subjective or objective cognitive decline, not considering healthy elderly subjects who are aware to have no difficulties or MCI subjects not completely aware of their difficulties. For this reasons, our data may not be representative of over-60 population. However, GR-2013-02356043 recruited a more representative community-based sample of general population than a clinical sample of people pertaining to clinics or departments to assess cognitive disorders.

Another major limitation of our study is that we obtained data through telephone interviews with people with MCI, who might present memory or judgment deficits that do not make them reliable witnesses. Unfortunately, the danger of contagion warranted against conducting face-to-face interviews. In any case, we excluded all GR-2013-02356043 participants who had obtained an MMSE score below 24 in their last visit, subjects with an Itel-MMSE score <17 and those unable to be interviewed according to their caregivers' judgment. Moreover, the data obtained from our sub-sample of participants with SCD are substantially consistent with those with MCI. This indicates that our participants with MCI may be adequately informative.

## Conclusions and Future Directions

People at increased risk of dementia underwent changes in their lifestyles that are potentially harmful for their cognitive and mental health. In particular, increased levels of sedentary lifestyle, which together with a less healthy diet led to weight gain in over a third of the sample, less social interaction, and greater engagement in passive recreational activities. However, even if, with the exception of productive leisure activities, increased smoke, alcohol or caffeine consumption, unhealthy diet, physical inactivity or time spent watching TV seem not be cross-sectionally related with mental health issues, it is possible that they have long-term effects. Further follow-ups will help us verify this hypothesis. The future directions should therefore focus on reducing loneliness, and on psychoeducational interventions, involving the patients and their caregivers to relieve anxiety associated with the onset of new respiratory symptoms, enhance awareness, healthy behaviors and reduce family conflicts, promoting the active listening, and mutual support between family members. Furthermore, one possible intervention is that of promoting a major awareness of the patients about the psychological help they may receive in case of need, since in our survey, elderly have never referred to *ad-hoc* services as the psychological help desk.

## Data Availability Statement

The raw data supporting the conclusions of this article will be made available by the authors, without undue reservation.

## Ethics Statement

Due to the exceptional situation, informed consent to the interview and the use of the data collected during the GR-2013-02356043 study was provided orally or by SMS or e-mail. The study and its amendment were approved by the Ethic Committee of the IRCCS Fondazione Santa Lucia of Rome.

## Author Contributions

SD, FF, and BF study project and questionnaire construction. SD database creation and data-analysis. FF, BF, AM, and SS data collection. SD, FF, BF, AM, and SS manuscript drafting. SD manuscript completion. All authors contributed to the article and approved the submitted version.

## Conflict of Interest

The authors declare that the research was conducted in the absence of any commercial or financial relationships that could be construed as a potential conflict of interest.
